# Atypical Presentation of Non-dominant Right Coronary Artery Occlusion: Precordial ST-Segment Elevation

**DOI:** 10.7759/cureus.90336

**Published:** 2025-08-17

**Authors:** Sinan Cerşit, Lütfi Öcal

**Affiliations:** 1 Cardiology, Health Sciences University, Kartal Koşuyolu Heart Training and Research Hospital, Istanbul, TUR

**Keywords:** atypical presentation of right coronary artery occlusion, coronary artery angiography, ecg-angiographic correlation, precordial st-segment elevation, st-segment elevation myocardial infarction (stemi)

## Abstract

This case report details a rare presentation of ST-segment elevation myocardial infarction (STEMI) in a 46-year-old male, highlighting an unusual electrocardiographic (ECG) pattern where proximal total occlusion of the right coronary artery (RCA) manifested with ST-segment elevation (STE) in both inferior leads (II, III, aVF) and, strikingly, anterior precordial leads (V1-V6). Typically, precordial STE is a hallmark sign of anterior myocardial infarction (MI), commonly linked to occlusion of the left anterior descending (LAD) coronary artery. This patient's ECG initially suggested a widespread myocardial insult, but subsequent coronary angiography definitively pinpointed a complete blockage in the proximal RCA, while the LAD and circumflex arteries were normal. This case should influence diagnostic reasoning by compelling clinicians to consider a wider differential diagnosis for STEMI and to avoid absolute reliance on classic ECG patterns. The diagnostic workup of such unusual cases should include clinical presentations with updated ECG criteria and integrated imaging algorithms such as comprehensive transthoracic echocardiography to ensure accurate diagnosis and timely reperfusion therapy.

## Introduction

Acute coronary syndromes (ACS), particularly ST-segment elevation myocardial infarction (STEMI), require accurate diagnosis for timely reperfusion, which is also crucial for outcomes in high-risk populations such as patients with right ventricular involvement, congestive heart failure, and human immunodeficiency virus-positive individuals [1-2]. Electrocardiography (ECG) is the primary diagnostic tool, providing immediate insights into myocardial ischemia. Conventionally, ST-segment elevation (STE) in precordial leads (V1-V6) signals an anterior myocardial infarction (MI), typically due to left anterior descending (LAD) coronary artery occlusion. Conversely, STE in inferior leads (II, III, aVF) points to an inferior MI, usually from right coronary artery (RCA) occlusion. These well-established ECG-angiographic correlations, often reinforced by reciprocal ST-segment depression, guide initial management [3].

However, the variable nature of coronary anatomy can lead to atypical ECG presentations, posing significant diagnostic challenges and potentially delaying vital reperfusion [1-4]. This report details a rare and instructive case of a 46-year-old male with diffuse STE across both inferior (II, III, aVF) and anterior precordial (V1-V6) leads. Despite this widespread STE, coronary angiography (CA) revealed a total proximal occlusion of the non-dominant RCA, with the left coronary arteries being patent. This striking discrepancy between ECG findings and the culprit vessel makes this case particularly compelling. This report will describe the clinical course, analyze the perplexing ECG-angiographic correlation, discuss potential underlying mechanisms for precordial STE in RCA occlusion, and underscore the critical importance of a vigilant, comprehensive diagnostic approach in STEMI, especially when faced with unusual electrocardiographic evidence.

## Case presentation

A 46-year-old male with a relevant medical history of smoking and hypertension presented to the emergency department experiencing several hours of acute chest pain, accompanied by profuse sweating and breathlessness. Despite these alarming symptoms, his initial physical examination yielded no remarkable findings, such as blood pressure, 120/65 mmHg; pulse, 75 beats per minute; respiratory rate, 16 breaths per minute; and oxygen saturation, 95% on room air, emphasizing the critical role of diagnostic tools in assessing his cardiac status.

**Figure 1:**
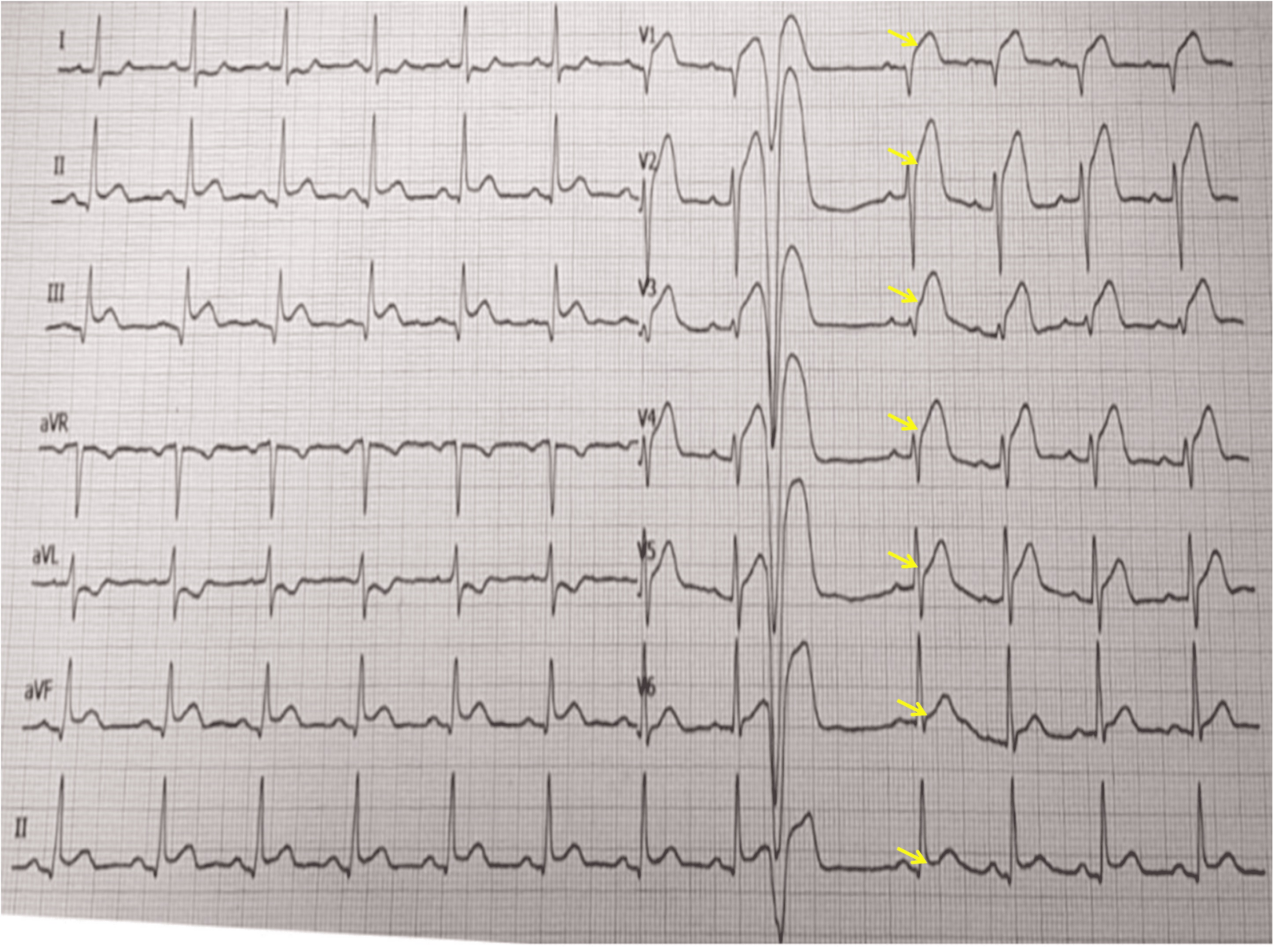
Initial ECG: precordial ST elevations

An immediate 12-lead ECG was performed, revealing a striking and complex pattern: significant STE in leads V1-V6, typically indicative of an extensive anterior MI, juxtaposed with concomitant STE in the inferior leads (II, III, aVF). Further complicating the picture, reciprocal ST-segment depression was noted in leads I and aVL. Given the prominent inferior STE and the clinical context, the initial working assumption leaned towards ischemia related to a RCA lesion, despite the unusual precordial changes (Figure 1).

To further assess cardiac function, a transthoracic echocardiography (TTE) was promptly conducted, which identified hypokinesis of the lower wall of the left ventricle, consistent with an inferior wall abnormality. Following this rapid diagnostic workup, the patient received standard dual antiplatelet therapy with 180 mg of ticagrelor and 300 mg of acetylsalicylic acid and was immediately transferred to the catheterization laboratory for definitive diagnosis and intervention.

**Figure 2:**
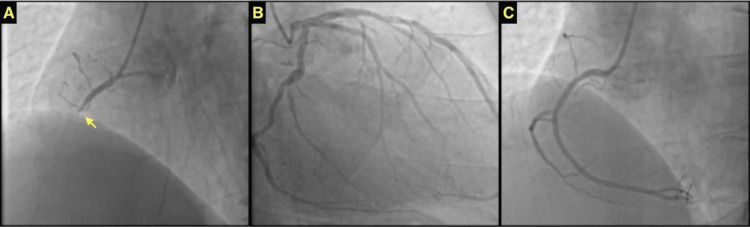
Coronary angiography A. Total proximal occlusion of the right coronary artery (RCA); B. Intact left anterior descending (LAD) and circumflex (Cx) coronary arteries. C. Normalization of blood flow after stenting.

Coronary angiography then provided the pivotal diagnostic clarity: it revealed a complete occlusion in the proximal portion of the RCA. Crucially, the LAD and circumflex coronary arteries were found to be entirely normal (Figure 2A, 2B). Subsequently, successful recanalization of the occluded RCA was performed, followed by the implantation of a 2.75x28 mm drug-eluted stent (DES), restoring myocardial blood flow (Figure 2C).

**Figure 3:**
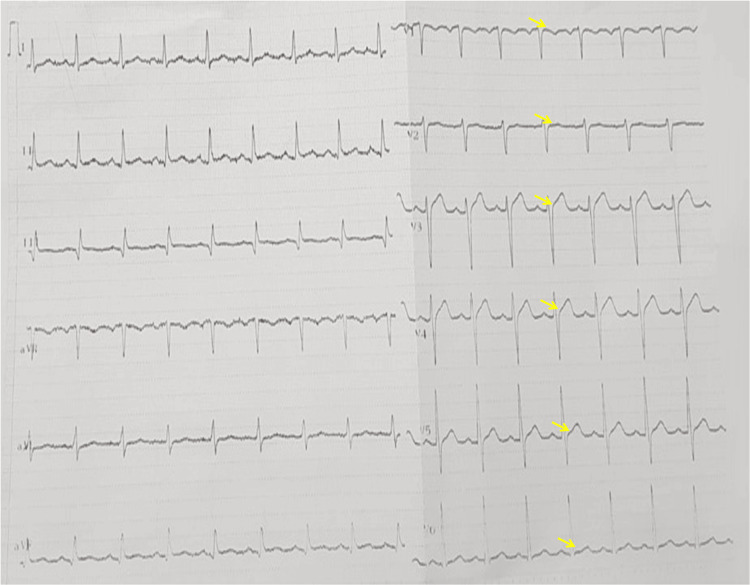
Post-procedural ECG: normalization of precordial ST segments

A post-procedural ECG impressively demonstrated full resolution of the STE across all affected leads, including V1-V6 (Figure 3). The patient's clinical condition stabilized rapidly. Three days later, he was discharged on double antiplatelet therapy, with follow-up TTE confirming a normalization of left ventricular contractile function.

## Discussion

The ECG signs for RCA occlusion demonstrated an increase in the amplitude of the ST segment in leads II, III, and aVF [1,5]. Sometimes the concomitant STE is found in the precordial and inferior leads, but rarely is the isolated STE in the precordial leads revealed [5,6]. ECG is important for the identification of the infarct-related artery in ACS, determination of the type of acute MI, making clinical decisions and treatment strategy, estimation of prognosis, as well as timely initiation of reperfusion therapy [7,8].

As a rule, STE in the precordial leads indicates occlusion of the LAD coronary artery [1,9]. However, in this case, there was an amazing phenomenon of STE in V1-V3 due to occlusion of the RCA. While RCA occlusions typically present with STE primarily in inferior leads, this instance of widespread precordial STE due to RCA involvement is uncommon. The phenomenon is attributed to the non-dominant nature of the RCA in this patient, which did not supply the posterior interventricular septum, thereby preventing the typical ST depression in V1-V3 and instead causing relative STE [9, 10]. In our patient, MI was present in both the anterior and inferior leads, based on non-dominant proximal occlusion of the RCA.

Our diagnostic approach was further supported by TTE, which revealed inferior wall hypokinesis. This finding aligned with the expected anatomical consequences of the RCA occlusion, even as the ECG presented a broader picture of ischemia. This highlights the value of multi-modal assessment in complex cases.

Some researchers proposed that premature ventricular contractions (PVC) may be caused by bradycardia due to RCA damage [10,11]. RCA damage may lead to bradycardia through ischemia and vagal tone, and this bradycardic state, in turn, may create a conducive environment for the development of PVCs through enhanced automaticity and triggered activity [10]. The current case was also presented by PVC on the background of the normal heart rate [11]. On post-MI treatment, particularly the role of beta-blockers, growing evidence suggests that their long-term use may not be as beneficial as previously believed [12].

## Conclusions

ECG guidance is of utmost significance for the prediction of the location of the culprit vessel. While STE in the anterior leads usually correlates with occlusions of the left coronary arteries, in rare cases, occlusion of the RCA can lead to such STE. This case serves as a valuable reminder for clinicians to consider atypical presentations of STEMI, emphasizing that not all precordial STE is indicative of LAD occlusion. The case also underscores the critical importance of integrating ECG findings with clinical presentation and advanced imaging, as conventional ECG-angiographic correlations can sometimes be misleading and may lead to a delay in accurate diagnosis and timely reperfusion therapy.
